# Supplementary material for: Biological Traits and Comprehensive Genomic Analysis of Novel Enterococcus faecalis Bacteriophage EFP6
Source: Microorganisms. 2024 Jun 14;12(6):1202. doi: 10.3390/microorganisms12061202 (PMC11206139; doi:10.3390/microorganisms12061202)
Supplement: Supplementary file 1 [file microorganisms-12-01202-s001.zip › microorganisms-3016025-supplementary.pdf]

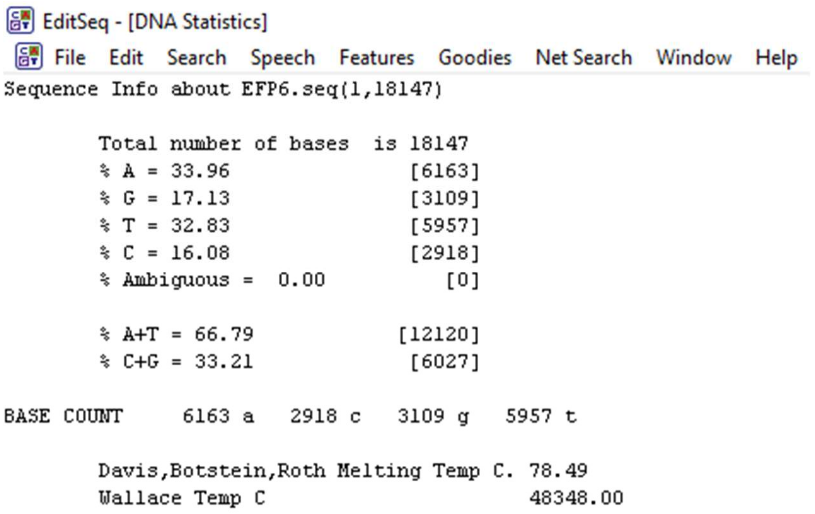


**Figure S1:** DNA statistics

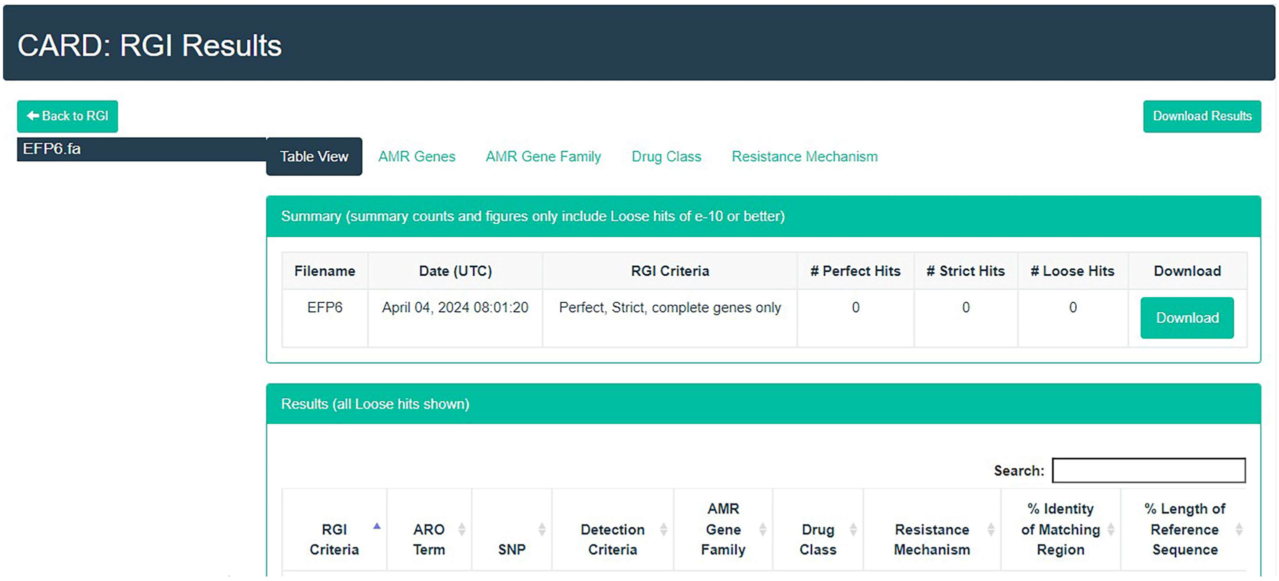


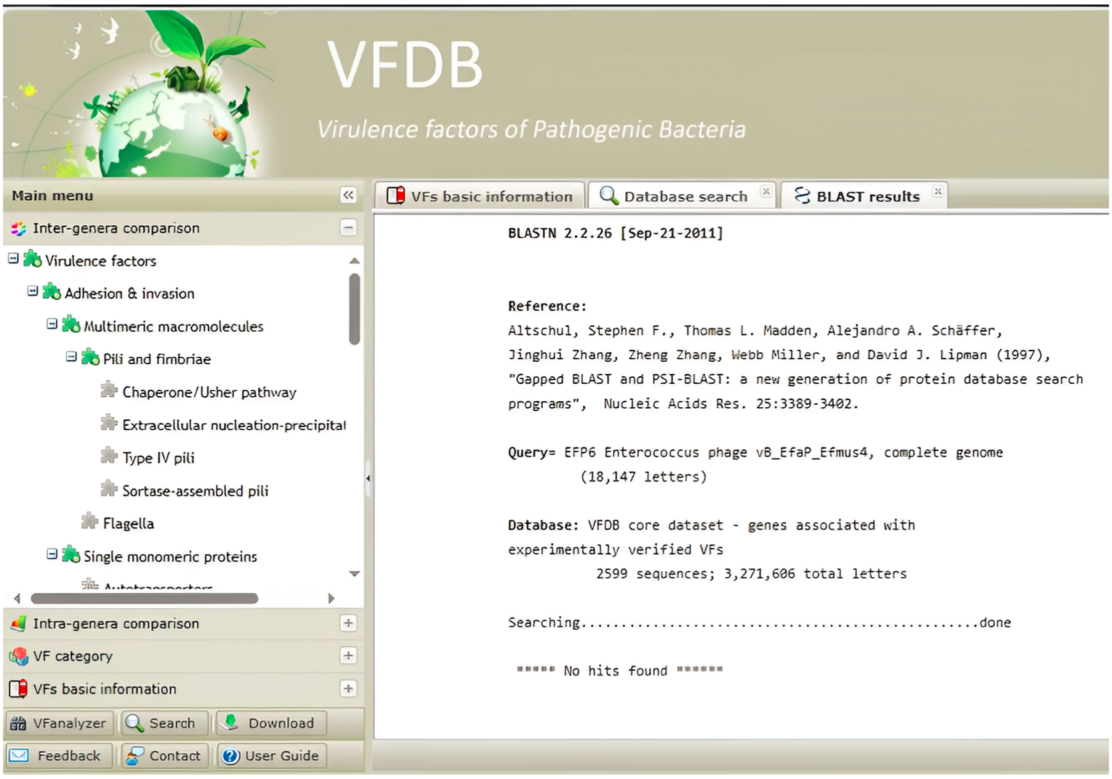

**Figure S2:** Genotypic Profiling (A) Antibiotic resistance genes in EFP6 and (B) identification of Virulence genes
